# Nurses job satisfaction identified by personal preferences and perceived saturation divergence: a comparative cross-sectional study in Czech hospitals between 2011 and 2021

**DOI:** 10.1186/s12912-023-01586-1

**Published:** 2023-11-10

**Authors:** Jiri Vevoda, Daniela Navratilova, Ondrej Machaczka, Petr Ambroz, Sarka Vevodova, Marco Tomietto

**Affiliations:** 1 Department of Humanities and Social Sciences, Faculty of Health Sciences, Palacký University Olomouc, Hněvotínská 976/3, Olomouc, 75 15 Czech Republic; 2 Department of Healthcare Management and Public Health, Faculty of Health Sciences, Palacký University Olomouc, Hněvotínská 976/3, Olomouc, 75 15 Czech Republic; 3 Department of Nursing, Midwifery and Health, Faculty of Health and Life Sciences, Northumbria University, Newcastle upon Tyne, NE1 8ST UK

**Keywords:** Hospital nurses, Job satisfaction, Herzberg´s theory, Personal preferences, Saturation, Euclidean Distance Model

## Abstract

**Background:**

The perception of the quality of care provided by the medical institution to patients is directly affected by the job satisfaction of nurses. The feeling of job satisfaction is caused besides other things by the subjective expectations of employees about what their work should provide them with in return. The aim of the study is to evaluate and compare job satisfaction of hospital nurses in the Czech Republic in 2011 and 2021 by identifying differences between their personal preferences and perceived saturation.

**Methods:**

The respondents are hospital nurses in the Czech Republic in 2011 and 2021. A developed questionnaire was used to determine the job satisfaction factors. The order of factors of personal preferences, perceived saturation and differences between them was compiled. For evaluation was used the Euclidean distance model that enables to capture the order and determine the significance given by the distance in which the factors are located.

**Results:**

At the top of personal preferences of hospital nurses, the factors salary and patient care are in the first two places with a similar distance. The salary factor is the most preferred by hospital nurses in both evaluated periods, and at the same time there is the greatest discrepancy between personal preferences and perceived saturation. By contrast, image of profession and working conditions were sufficiently saturated by the employer in both periods, but nurses do not significantly prefer these factors.

**Conclusions:**

The salary and patient care (i.e. the mission of the nurse’s work itself) are at the top of personal preferences of hospital nurses, with an exclusive position among other factors. We consider it important that the hospital management emphasizes them in the management of hospital nurses. At the same time, the patient care is perceived by the hospital nurses as one of the most saturated factors - in contrast to salary, which is located at the complete opposite pole as the least saturated factor and therefore emerges from the mutual comparison as the factor with the greatest degree of divergence. The stated conclusions are valid for both compared periods. New method of data evaluation was successfully tested.

## Introduction

Healthcare organisations are  facing a growing shortage of healthcare workers (HCWs) [1, 2]. The COVID-19 pandemic has revealed more than ever how crucial the area of patient care held by nurses is for the functioning of society and the state as a whole [3]. Tackling these trends is crucial to providing quality care and patient safety. While educating more HCWs and nurses is important, it is also strategic to retain the current workforce and decrease the turnover intention and the intention to leave the profession [4]. Job satisfaction is a core concept to look at, when planning organisational interventions to retain HCWs, decrease turnover intentions [5] and provide quality care [6]. Most studies realized in various countries clearly confirm that the level of job satisfaction significantly affects the turnover of nurses [7–10] and predicts the likelihood of burnout [11]. Experts also examine the intention itself, i.e., the mere considerations of quitting and ideas about the possibility of looking for alternative employment [12, 13]. Increased nursing turnover leads to reducing the quality of health care, higher rates of errors, morbidity and mortality, and also means financial losses for health care providers [14–16]. And conversely, the stability of the health workforce brings economic and other benefits to health care providers and caregivers [17]. I.e., the job satisfaction of nurses directly affects the perception of the quality of care provided by the medical institution to patients [18]. It is therefore in the interest of the management of health care providers to increase job satisfaction and strengthen the motivation of nurses.

One of the factors that significantly affect the job satisfaction of employees is remuneration [19, 20]. The published average salaries of nurses include overtime hours. Especially for economic reasons, nurses often practice so-called dual practice, i.e. simultaneous employment in the public and private sectors [21]. Overtime is a common occurrence in the nurse’s profession [22, 23] - unlike some occupations, which also affect the average wage level. This fact makes difficult to compare the average salary of nurses with the stated national average wage. In the Czech Republic the health and medical professions have been significantly underestimated for many years. The situation changed after 2015, when wages in health care began to rise significantly [24]. The average monthly wage of nurses increased by 71% in five years to the current level which is 1.14 times higher than the average wage in the Czech Republic [25].

Besides the increase in financial remuneration, there were other significant changes regarding the situation of nurses in the Czech Republic during the monitored period. Czech healthcare is facing an unfavorable trend regarding the aging of nurses and their overall shortage in healthcare practice [26]. In the monitored period, there was a shift in the age structure of general nurses in the Czech Republic. While in 2011 three age groups of 35–49 years prevailed fairly evenly [27], in 2017 most nurses fell into the age category of 40–44 years and in 2021 most nurses were aged 43–47 years [28]. Both of these trends (aging and decline) lead to a forecast that by 2050 assumes a predominant age structure of 55–59 years with a total shortage of 30,000 nurses in healthcare practice against the current state [29]. It is therefore obvious that the workload of nurses in the Czech Republic is continuously increasing [30]. Also, the impact of the COVID-19 pandemic on the Czech healthcare system and hospitals had a profound effect on nurses and their perception of their work environment [31].

Long-term studies by the Centre for Public Opinion Research show that the profession of nurse has been ranked 3rd in the most prestigious occupations according to the opinion of the Czech population in the last ten years - right after a medicine doctor and a scientist [32]. In parallel, prestige is one of the factors influencing subjectively perceived job satisfaction [33]. The question is to what extent the positive social status and social demand of this profession from the point of view of the external environment (society) is reflected in the perception of the holders of this profession themselves, i.e. whether the nurses themselves are aware of the desirability of their profession, including the social requirements that are placed on the performance of their profession [34], and the extent to which this perception affects overall job satisfaction. Job satisfaction is significantly affected not only by the nature of the work as such, but also by the subjective expectations of employees about what their work should provide them with in return [35]. In other words, job satisfaction occurs when the experience in the work environment conforms to the value of the employee [36]. Job satisfaction is then considered as a perception that an individual has about the own job [37].

One of the most recognized models to explore and understand job satisfaction is Herzberg´s theory [33]. Herzberg distinguishes two types of motivational factors: satisfiers (motivators), intrinsic factors which are a source of job satisfaction and motivation (this includes success, recognition, work itself, responsibility, ability to progress), and dissatisfiers (hygiene factors), extrinsic factors whose absence or poor quality is a source of dissatisfaction, while their presence does not have a direct effect on satisfaction (this includes factors such as working conditions, salary, peer relations, administrative policy and supervision) [38]. Herzberg used this model to explain that an individual at work can be satisfied and dissatisfied at the same time, because the two sets of factors work in separate sequences. In other words, that job satisfaction depends on both internal and external factors. For example, hygiene factors cannot increase or decrease satisfaction, they can only affect the level of dissatisfaction. The application of this theory to the study of job satisfaction also allows knowing the value preferences of the employee and their arrangement in the so-called personal value ladder. Herzberg’s two-factor theory has been applied in many studies dealing with job satisfaction not only in healthcare [33, 39–42]. The results suggest that remuneration, employee engagement, and the work environment are important predictors of job satisfaction [43]. An equally important factor is the emotional intelligence, empathy, and communication skills [44], as well as appreciation from managers and colleagues [39, 45].

The aim of the study is to evaluate and compare job satisfaction of hospital nurses in the Czech Republic in 2011 and 2021 by identifying differences between their personal preferences and perceived saturation using Euclidean Distance Model (EDM).

Most studies devoted to job satisfaction only deal with nurses’ personal preferences [18]. Our research moves this view to the next level. Along with personal expectations, we are also interested in the degree of saturation of individual factors by the employer. Subsequently, we compare both of these views, thereby obtaining a clear ranking of the most and least different factors of nurses’ job satisfaction. The tool we use to interpret the outputs is also unconventional. We are interested in EDM, which captures the mutual distance of individual factors that also determines the significance of these factors.

## Methods

### Settings

This study was conducted in hospitals in the Czech Republic. A cross-sectional study involving questionnaire survey was realized in both 2011 [46] and 2021. In 2011, the research was carried out from November to December, and in 2021 from September to October. In both research periods, the entire territory of the Czech Republic was covered. The survey itself was personally carried out by experienced interviewers, specially trained for the purpose of this research. The research was conducted by the interviewer using CATI (Computer Assisted Telephone Interview) survey technique. Before starting the verbal interview itself, all participants were informed about the purpose of the research, the planned use of the results, the author of the study, the method of storage and further handling of the provided answers and about the method of ensuring the anonymity of the provided answers. This introduction was followed by a direct question from the interviewer, whether the interviewee agrees to his participation in the research and whether it is possible to proceed to the interview itself. The informed consent of the interviewee was recorded. The research was subject to the approval of the Ethics Committee of the Faculty of Health Sciences Palacky University Olomouc. The data was handled in accordance with the strict rules of the General Data Protection Regulation (GDPR).

### Respondents

The respondents were only nurses working in hospitals in the Czech Republic. The inclusion criteria were the finished adaptation process and provided informed consent and expressed willingness to participate in the survey. Nursing students and nurses working part-time in these hospitals were excluded. Research participants were selected according to: region of the hospital; age of HNs; and gender of HNs. The aim of this selection is to reach such a sample of respondents as would correspond to the composition of HNs in the Czech Republic according to the National Health Information System. Respondents were selected at following random using quotas:

(A) In 2011 the register of healthcare workers had not yet been established in the Czech Republic, the number of HNs in individual regions was taken as the basic indicator of representativeness when constructing the sample set. Within the research, HNs from all regions were approached, while their representation corresponds to the structure of the basic set. It can be stated that the deviation from the base set in 2011 did not exceed 0.3%. The second characteristic for which representativeness was monitored was the age of the HNs. The parameters of the basic set were derived from the data presented in the Labor Market Forecast 2009 - Healthcare Professionals [47]. The deviation from the structure of the base set from the point of view of this second parameter amounted to a maximum of 0.4%. A total of 2,223 HNs were contacted for a questionnaire survey. Response rate was 89.6%.
(B) In 2021, the sample of HNs was constructed on the basis of indicators given by the basic group of HNs in the Czech Republic [48] according to the National Health Information System (was established by law in 2017 and therefore the data base could not serve as a basis for providing data on the structure of HNs for research conducted in 2011). The region and age of HNs were determined as indicators of representativeness. Considering the determined quotas, hospitals were first randomly selected, followed by HNs meeting the gender and age indicators. It can be stated that, compared to the age distribution of the base set, the deviation of the research set does not exceed 0.5%. From the point of view of the regions, the deviation of the research set does not exceed 0.3% compared to the base set. Interviewers approached a total of 670 HNs to questionnaires. The lower number of respondents compared to 2011 was caused by the COVID-19 pandemic, which hit the Czech Republic hard in 2021, as well as the rest of the world. This period meant a great strain on healthcare workers. Compared to 2011, the accessibility and the possibility of HNs to participate in research was significantly limited. Response rate was 88.4%. As in the previous survey, respondents were selected at random using quotas. Detailed description of respondents from both evaluated periods is shown in Table 1. There is a different size of the samples of respondents in the monitored years. Nevertheless, the research set in 2011 was considerably oversized due to the size of the base set and the composition of both samples reflects the composition of the basic population of hospital nurses in given years, therefore it can be considered sufficiently representative.

**Table 1:** Description of respondents

	2011		2021	
	N	%	N	%
**Total**	1992		592	
**Sex**				
Female	1913	96.0	571	96.5
Male	79	4.0	21	3.5
**Education**				
Graduated	445	22.3	341	57.6
Post-secondary	486	24.4	57	9.6
Secondary	1022	51.3	194	32.8
Other	39	2.0	0	0
**Managerial position**	361	18.1	60	10.1
**Average age**	42 (years)		43,6 (years)	

### Instrument a measurement

A developed questionnaire was used to determine the motivation and job satisfaction of hospital nurses, the methodological framework of which is Herzberg’s two-factor motivational theory [38]. The motivational and hygienic factors defined by Herzberg became the basis for compiling value scales. Subsequently, the scales and factors were consulted with experts from practice (HNs, managers) and were adjusted to suit the Czech socio-cultural and work environment. The focus group had 8 members, a total of 5 meetings took place. Outputs in the form of 5 additional factors affecting job satisfaction were determined. Factors from Herzberg’s theory and adjusted factors included in the questionnaire are shown in Table 2.

**Table 2:** Factors assessed in the questionnaire

**Factors from Herzberg’s theory**
Relationships with co-workers
The possibility of career growth
Prestige of the profession
Adequacy of salary or wage
Recognition of personal work result from both superior and colleagues
Job security (a sense of security, the employee does not feel threatened by e.g. dismissal)
The work itself (interesting, meaningful tasks, challenges)
Working conditions (safe and healthy work environment, supporting work content)
Corporate climate (understandable, fair company policies)
**Additional factors**
Cooperation with other professions (mutual cooperation and support)
The possibility of using modern technical and technological equipment
The possibility of further vocational training
The image of the employer
Information provided (intelligibility of tasks, availability of databases)
Non-monetary benefits (employee/social benefits)

These individual work environment factors are sorted by HNs by forced selection from 1 (most important factor) to 16 (least important factor). The selection is made separately for personal preferences and for perceived saturation, i.e., how they perceive the fulfilment of these factors by their employer - the hospital.

### Analysis

The sample was evaluated and compared as a whole, in individual years – 2011 and 2021. For each year, the order of factors of personal preferences, perceived saturation and divergence between them was compiled. IBM SPSS 19.0 Base was used to evaluate the data. The Euclidean distance model (EDM) was used for evaluation of order and distance between individual factors of the working environment [49, 50]. The statistical test one-dimensional EDM is one of the tests of cluster analysis. Based on the mutual similarity of factors, the test classifies them into groups of clusters. The used one-dimensional EDM is able to capture both the order and determine the distances in which the individual factors are located. If the factors are grouped into one space, it means their considerable homogeneity; if, on the contrary, they are scattered along the axis, it means their heterogeneity. By their distance from others, they also determine their significance (the so-called emotional range). If the factors are far apart, then their importance to the neighbouring factor and, of course, to other factors grows [49].

The biggest negative divergences have been identified, which represent a discrepancy between personal preferences and their perceived fulfilment by the employer (saturation). Furthermore, the individual years were compared with each other.

## Results

**Fig. 1:**
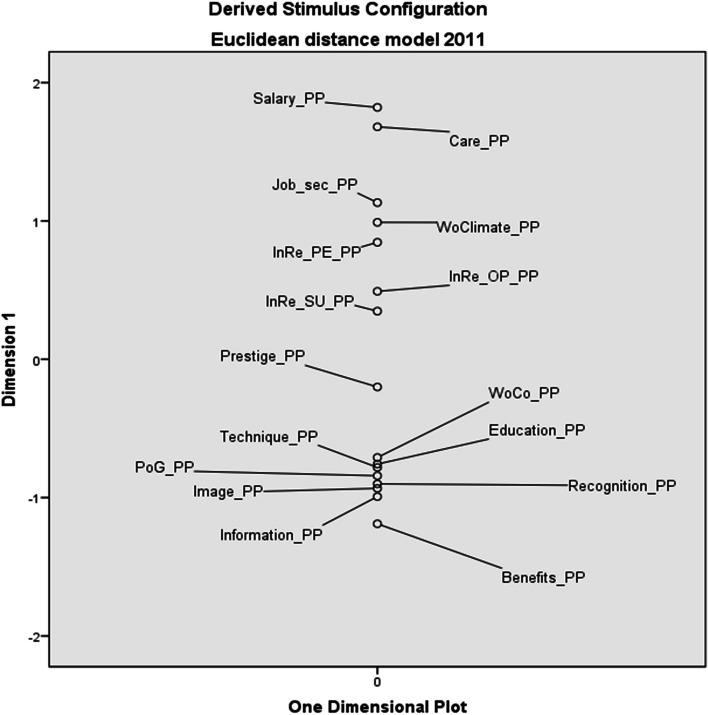
Euclidean distance model of personal preferences of work environment factors – 2011 (*PP* personal preferences, *Job_sec* job security, *WoClimate* working climate, *InRe_PE* internal relations with co-workers, *InRe_OP* internal relations with other professions, *InRe_SU* internal relations with superior, *WoCo* working conditions, *PoG* possibility of career growth)

Using the EDM, it was found that in terms of subjectively perceived preference of work environment factors, HNs in 2011 most preferred the factors salary (Salary_PP), patient care (Care_PP) and job security (Job_sec_PP). The least preferred factor were social benefits (Benefits_PP) – see Fig. 1.

**Fig. 2:**
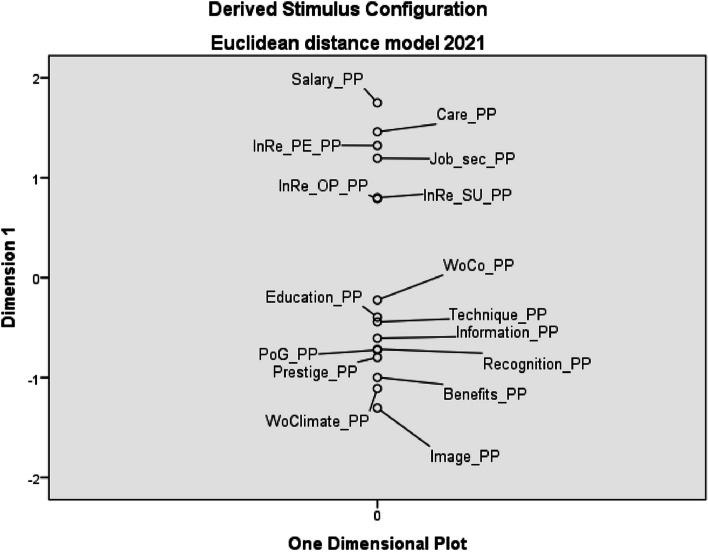
Euclidean distance model of personal preferences of work environment factors – 2021 (*PP* personal preferences, *InRe_PE* internal relations with co-workers, *Job_sec* job security, *InRe_OP* internal relations with other professions, *InRe_SU* internal relations with superior, *WoCo* working conditions, *PoG* possibility of career growth, *WoClimate* working climate)

In 2021, salary and patient care were again identified as the most preferred factors. In third place in terms of preferences turned out to be internal relations with co-workers (InRe_PE_PP), which in 2011 were in the order of preferences up to 5th place. HNs identified image of their profession (Image_PP) as the least preferred factor in 2021. The most significant change occurred in the working climate factor (WoClimate_PP), which the HNs mentioned in 2011 as their 4th preference. In 2021, however, this factor fell to the second least preferred factor (see Fig. 2).

**Fig. 3:**
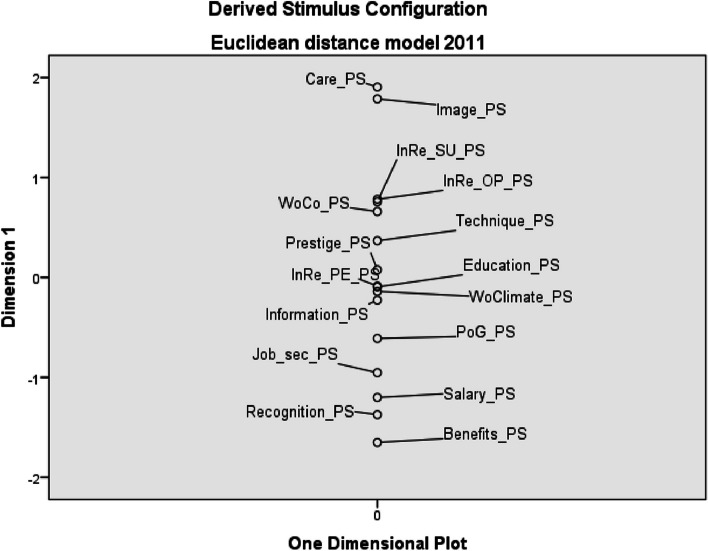
Euclidean distance model of perceived saturation of work environment factors – 2011 (*PS* perceived saturation, *InRe_SU* internal relations with superior, *InRe_OP* internal relations with other professions, *WoCo* working conditions, *InRe_PE* internal relations with co-workers, *WoClimate* working climate, *PoG* possibility of career growth, *Job_sec* job security)

In terms of subjectively perceived saturation of factors by the employer, in 2011 HNs considered the most saturated factor to be patient care (Care_PS), image of the profession (Image_PS) and internal relations with superior (InRe_SU_PS). The least saturated factor were social benefits (Benefits_PS), non-monetary recognition of personal work results (Recognition_PS) and salary (Salary_PS) – see Fig. 3.

**Fig. 4:**
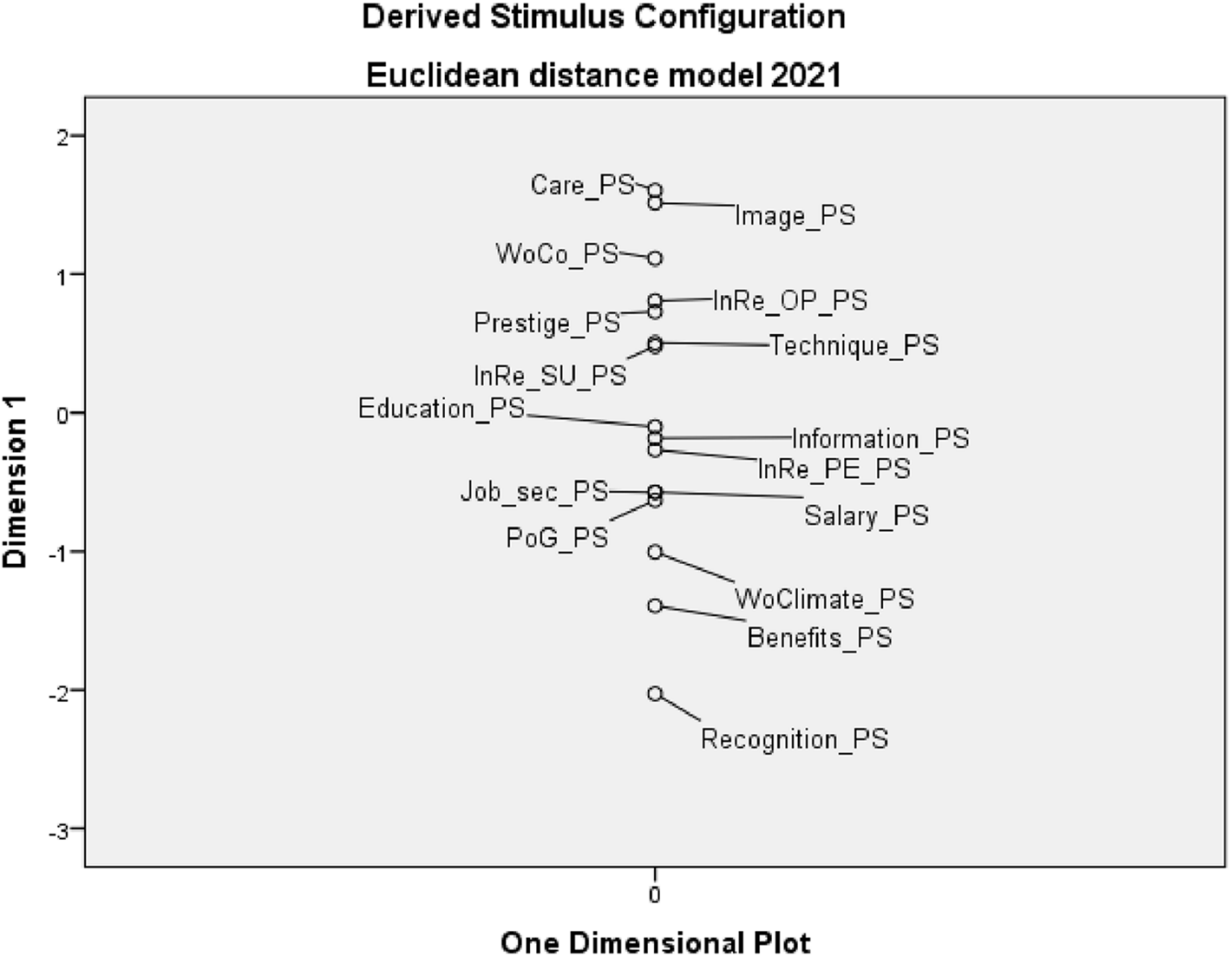
Euclidean distance model of perceived saturation of work environment factors – 2021 (*PS* perceived saturation, *WoCo* working conditions, *InRe_OP* internal relations with other professions, *InRe_SU* internal relations with superior, *InRe_PE* internal relations with co-workers, *Job_sec* job security, *PoG* possibility of career growth, *WoClimate* working climate)

In 2021, non-monetary recognition of personal work results, social benefits and the workplace climate are perceived as the least saturated factors. The perception of salary saturation has changed slightly, the position of which has “improved” by two places compared to the period ten years ago (see Fig. 4).

**Fig. 5:**
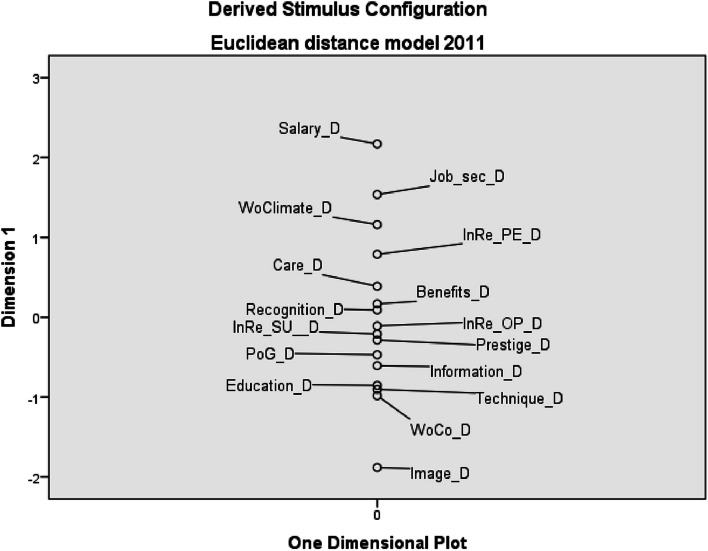
Euclidean distance model of divergence between personal preferences and perceived saturation – 2011 (*D* divergence, *Job_sec* job security, *WoClimate* working climate, *InRe_PE* internal relations with co-workers, *InRe_OP* internal relations with other professions, *InRe_SU* internal relations with superior, *PoG* possibility of career growth, *WoCo* working conditions)

The biggest divergence between the preference of work environment factors and their saturation was found in 2011 for the factors salary (Salary_D) and job security (Job_sec_D). These factors are highly valued by nurses in terms of importance but are also the least saturated by the employer. In contrast, image of profession (Image_D), working conditions (WoCo_D) and the possibility of using modern technical and technological equipment (Technique_D) were sufficiently saturated by the employer in 2011, but nurses do not significantly prefer these factors (see Fig. 5).

**Fig. 6:**
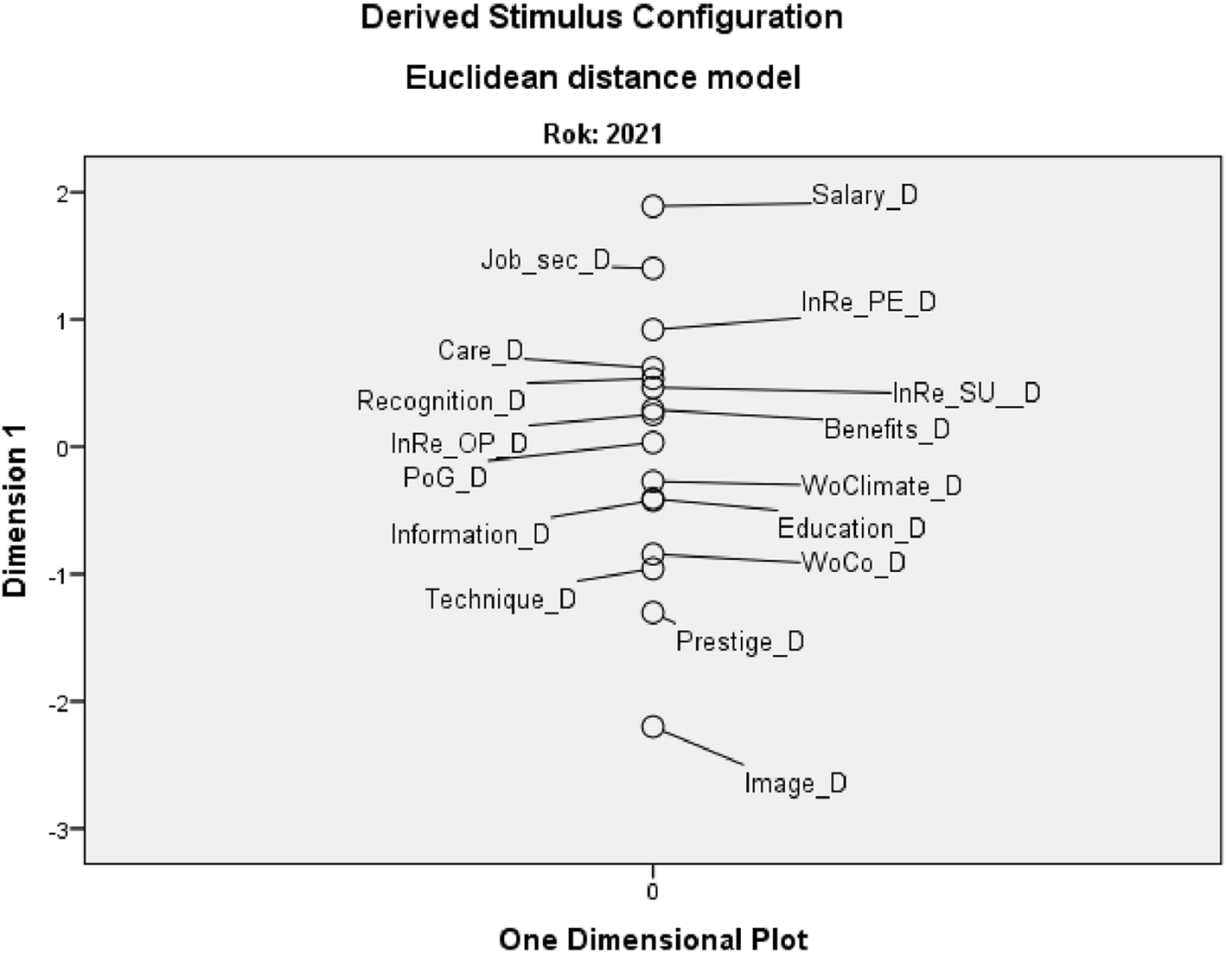
Euclidean distance model of divergence between personal preferences and perceived saturation – 2021 (*D* divergence, *Job_sec* job security, *InRe_PE* internal relations with co-workers, *InRe_SU* internal relations with superior, *InRe_OP* internal relations with other professions, *PoG* possibility of career growth, *WoClimate* working climate, *WoCo* working conditions)

In 2021, the order of factors with the most significant divergence between HNs’ preference and saturation by employer remained almost the same. As in 2011, salary remains the factor with the strongest negative divergence. Thus, it is still the factor with which the HNs are least satisfied and where they perceive the strongest divergence between their personal priorities and its saturation. Similarly, but not with such a strong divergence, job security appears in both examined periods. From the opposite point of view (slightly preferred by the HNs, but well-saturated factors by the employer) the order of factors did not change much compared to 2011 - again there is the greatest divergence in image of the profession and the possibility of using modern technical and technological equipment, only the working conditions were replaced by prestige of profession in public (Prestige_D) - see Fig. 6.

## Discussion

As the first, our research presents the use of an original method for evaluating job satisfaction by identifying the divergence between preference and saturation of selected factors determining the job satisfaction of nurses in Czech hospitals. By comparing the obtained data, this method enables to compile a clear ranking of the most and least diverging factors.

The results of the study clearly show that salary (together with patient care) is at the top of personal preferences of HNs (see Figs. 1 and 2), but at the same time it is the least saturated factor (see Figs. 3 and 4). This fact is also confirmed by the authors of other studies, e.g. in Greece or in the USA (New Hampshire) [51, 52]. In our research, salary was a factor with the greatest divergency in both evaluated periods. It is interesting that this divergence did not change in Czech Republic, although there has been a significant increase in HNs’ salaries in monitored period.

The second most preferred factor was patient care, but, unlike salary, it was the most saturated factor. The salary and patient care are heterogeneous factors, with an exclusive position among other factors. From the point of view of Herzberg’s theory, it is one satisfactor (salary) and one motivator (patient care). An interesting perspective at the question of salary preference and the importance of patient care in relation to nurses’ job satisfaction is offered by Jezorska [53], who together with nurses’ job satisfaction also focused on the rate of burnout syndrome: the salary factor may seem to be a crucial factor, due to its prioritization of HNs and the perceived insufficient fulfilment, its insufficient saturation alone does not lead to emotional exhaustion. Her research showed connection between burnout syndrome and salary preference over the preference for motivators (such as patient care or team cooperation) that dominated nurses who did not show signs of burnout syndrome. Unlike the salary factor, the insufficient saturation of the factors climate at the workplace, working conditions, cooperation with co-workers or non-monetary recognition of work results has a fundamental influence on the occurrence of burnout syndrome. Non-monetary rewards of work or workplace climate have proven to be more effective in preventing burnout syndrome - than satisfaction with salary.

A similar insight into nurses’ satisfaction can be found in the paper of Roth [54] who dealt with factors that could help reduce nurse turnover in Germany. As in our research, in work of Roth, the satisfaction of nurses is viewed from a double perspective: what the nurses themselves expect from the profession (in Roth´ research, so-called pull factors) and what factors are saturated by employers (in Roth´ research, on the contrary, what factors are absent – so called push factors). In Roth´ research, salary is mentioned as a pull factor, i.e. a factor whose improvement could lead to a decrease in the turnover of nurses, or to decrease their dissatisfaction. Image, prestige of profession (in Roth´ research called professional pride) and recognition of nursing are perceived by German nurses as very important. The research shows that in Germany, unlike in the Czech Republic, the image of the nursing profession is at a low level. Perhaps that is why this issue is more important for German nurses than for Czech nurses, for whom the issue of image is not so important, although Czech society perceives the profession of nurse as the third most prestigious profession. For German nurses, the salary factor itself is closely related to the image and prestige of the nursing profession as such, rather than to the financial income, which may be due, among other things, to the higher standard of living in Germany compared to the Czech Republic.

As for the prestige factor, there has been a significant shift to highly saturated factors between 2011 and 2021. The placement of the nurse profession in third place in the ranking of the most prestigious professions in the Czech Republic from the point of view of the general public [32] could be the reason of this shift. Unfortunately, prestige is a factor that occupies lower ranks in terms of personal preferences of HNs and thus does not have a significant effect on their job satisfaction.

On the contrary, internal relations with other professions as physicians, paramedical staff, etc., occur between the preferred and at the same time saturated factors in both examined periods. As confirmed by the divergence graph, internal relations with other professions are one of the factors that contribute to job satisfaction of nurses. A similar conclusion reached, among others, Zangaro and Soeken [55]. A slight shift over the last ten years can also be seen in internal relations with co-workers. According to research, this factor is perceived by nurses in 2021 as less saturated than it was ten years ago, while a higher degree of preference remains. This increases the divergence of this factor. This shift could have been influenced by the covid situation and the related workload at selected workplaces [56] as well as the significant aging of nurses and their general shortage in the Czech Republic, as mentioned in the Introduction.

The placement of the nurse profession in third place in the ranking of the most prestigious professions in the Czech Republic from the point of view of the general public affected the HNs’ saturation ladder, within which the prestige factor shifted to highly saturated factors. Unfortunately, this is a factor that occupies lower ranks in terms of personal preferences of HNs and thus does not have a significant effect on their job satisfaction. On the contrary, internal relations with other professions as physicians, paramedical staff, etc. (InRe_OP), occur between the preferred and at the same time saturated factors in both examined periods. As confirmed by the divergence graph, internal relations with other professions are one of the factors that contribute to job satisfaction of nurses. Zangaro and Soeken, among others, reached a similar conclusion [55]. A slight shift over the last ten years can also be seen in internal relations with co-workers (InRe_PE). According to research, this factor is perceived by nurses in 2021 as less saturated than it was ten years ago, while a higher degree of preference remains. This increases the divergence of this factor. The question remains whether this shift could have been influenced by the COVID-19 situation and the related workload at selected workplaces [56]. Among the least saturated factors, recognition by the direct superior (Recognition_PS) appears in both periods. The importance of praise and recognition is also emphasized by Ivanova [34] who states that the most important for nurses is praise from the patient or their family, followed by the recognition from those who professionally understand their work. A number of studies confirm the irreplaceable role of management in the process of setting conditions that lead to improved job satisfaction of nurses [45, 57, 58]. It is therefore one of the challenges for management to direct their measures to increase the saturation of those factors that occupy higher ranks in the preferential ranking of HNs.

If we distinguish between intrinsic and extrinsic motivation when looking at motivational factors, then theoretical and practical works agree that extrinsic motivational factors are not as effective as intrinsic factors [38, 59–61]. But our research shows the opposite: the most important factors from the point of view of HNs are salary and relationships at the workplace. This may be due to the already mentioned long-term salary undervaluation of the nursing profession (the situation has only changed in recent years) and also the still persistent perception of nurses by doctors as a less valuable member of the team. Here, however, we are already encountering the limits of quantitative research, within which reality can be reduced. Undoubtedly, this is an interesting stimulus for further research using the methodology of qualitative research, which would focus on international comparisons and find out the causes of the mentioned discrepancy.

Limitations of the study relate to the possibility of comparison with other research. Researchers often use different methods of data evaluation or techniques, information collection or different instruments to measure job satisfaction. This diversity of instruments used means a problem in comparing the findings of research studies. International comparison of the results of studies that use standardized questionnaires also appears to be difficult, as the cultural or political background of the countries where the research is conducted is different. As another limitation of the work may appear different size of the sample of respondents in the monitored years. Nevertheless, the results are valid at a comparable level of significance. The research set in 2011 was considerably oversized due to the size of the base set. Besides that, the composition of both samples reflects the composition of the basic population of HNs in given years and therefore it can be considered sufficiently representative. A certain limitation of the research could be the specific work environment during the COVID-19 pandemic. This fact must be considered when interpreting the results of the comparison of both periods. Subsequent research comparing the job satisfaction of nurses in the post COVID-19 period appears to be another challenge. This opens up space for a future unique comparison of the situation before COVID-19, during the ongoing pandemic and the period after COVID-19.

The benefit of the present study is a twofold comparison of work process factors: first according to the personal preferences of the HNs and then according to the saturation factors provided by the employer (hospital), as the HNs subjectively perceive it. The EDM method was successfully tested to evaluate the obtained data. Its use is original in the context of other studies researching job satisfaction.

## Conclusions

Our research clearly showed that salary is a factor that is preferred by HNs in both evaluated periods, and at the same time there is the greatest divergency between personal preferences and perceived saturation. This divergency persists even in 2021, when the remuneration of HNs was already at a completely different level than ten years ago. Patient care – i.e., the content of work itself, is the factor that HNs also prioritize the most in the personal value ladder, but, unlike salary, they perceive it as the most saturated factor, together with the image of the profession. We consider it important that the hospital management emphasizes them in the management of HNs.

Together with the salary and the content of the nurse’s work, the quality of interactions between nurses and other professions plays a significant role in influencing nurses’ job satisfaction.

Recognition by the direct superior stands out as one of the less saturated factors present in both research periods.

Based on our research, it can be concluded that creating a work environment in which HNs will be satisfied is one of the key elements to stabilize them and reduce the negative impacts associated with high turnover of nurses.

## Data Availability

The datasets used and analysed during the current study are available from the corresponding author on reasonable request.

## Abbreviations

CATI: Computer assisted telephone interview
COVID-19: Coronavirus disease of 2019
D: Divergence
EDM: Euclidean distance model
GDPR: General data protection regulation
HCWs: Healthcare workers
HN/HNs: Hospital nurse/Hospital nurses
InRe_OP: Internal relations with other professions
InRe_PE: Internal relations with co-workers
InRe_SU: Internal relations with superior
Job_sec: Job security
MDS: Multidimensional scaling analysis
PoG: Possibility of career growth
PP: Personal preferences
PS: Perceived saturation
WoClimate: Working climate
WoCo: Working conditions
